# Identification of Small Molecule Inhibitors of Pre-mRNA Splicing*[Fn FN2]

**DOI:** 10.1074/jbc.M114.590976

**Published:** 2014-10-03

**Authors:** Andrea Pawellek, Stuart McElroy, Timur Samatov, Lee Mitchell, Andrew Woodland, Ursula Ryder, David Gray, Reinhard Lührmann, Angus I. Lamond

**Affiliations:** From the ‡ Centre of Gene Regulation and Expression and; the § Drug Discovery Unit, University of Dundee, Dundee DD1 5EH, Scotland, United Kingdom and; the ¶ Max Planck Institute for Biophysical Chemistry, 37077 Göttingen, Germany

**Keywords:** High Throughput Screening, RNA, RNA Splicing, Small Molecule, Spliceosome, DDD00107587, Madrasin

## Abstract

Eukaryotic pre-mRNA splicing is an essential step in gene expression for all genes that contain introns. In contrast to transcription and translation, few well characterized chemical inhibitors are available with which to dissect the splicing process, particularly in cells. Therefore, the identification of specific small molecules that either inhibit or modify pre-mRNA splicing would be valuable for research and potentially also for therapeutic applications. We have screened a highly curated library of 71,504 drug-like small molecules using a high throughput *in vitro* splicing assay. This identified 10 new compounds that both inhibit pre-mRNA splicing *in vitro* and modify splicing of endogenous pre-mRNA in cells. One of these splicing modulators, DDD00107587 (termed “madrasin,” *i.e.* 2-((7methoxy-4-methylquinazolin-2-yl)amino)-5,6-dimethylpyrimidin-4(3*H*)-one RNAsplicing inhibitor), was studied in more detail. Madrasin interferes with the early stages of spliceosome assembly and stalls spliceosome assembly at the A complex. Madrasin is cytotoxic at higher concentrations, although at lower concentrations it induces cell cycle arrest, promotes a specific reorganization of subnuclear protein localization, and modulates splicing of multiple pre-mRNAs in both HeLa and HEK293 cells.

## Teaser

**Background:** There is a need for new small molecule pre-mRNA splicing inhibitors as biotools.

**Results:** High throughput screening resulted in the identification of small molecule splicing inhibitors that are active *in vitro* and in cells.

**Conclusion:** New small molecules for studying pre-mRNA splicing *in vitro* and in cells are identified.

**Significance:** Small drug-like molecules are identified that modulate splicing *in vitro* and in cells.

## Introduction

The removal of introns from pre-mRNA is an essential step in gene expression in eukaryotes. Deep sequencing data have shown that >95% of all human genes are spliced, often in a developmental, tissue-specific, and/or signal transduction-dependent manner (1). Pre-mRNA splicing is mediated by the spliceosome, a large RNA-protein complex that in humans consists of five snRNAs (U1, U2, U4, U5, and U6) and numerous proteins (2). The spliceosome coordinates the two separate transesterification reactions required for intron removal and the completion of the splicing cycle. The splicing of pre-mRNA takes place in the cell nucleus and involves the stepwise assembly of a separate spliceosome complex on each intron in a pre-mRNA transcript. In most, if not all cases, spliceosomes assemble on nascent transcripts while transcription of the gene is still in progress.

The maintenance of high fidelity pre-mRNA splicing is important to allow accurate protein expression. Point mutations affecting pre-mRNA sequences involved in splice site recognition and splicing are thought to be responsible for ∼15% of all inherited human diseases (3, 4). As well as its essential role in removing introns to permit protein expression, different patterns of alternative splicing allow the creation of multiple mRNAs encoding different proteins from a single gene. Alternative splicing is also often disrupted in many forms of disease, including cancer. For example, this can be due to either altered expression or mis-localization of RNA-binding proteins, resulting in changes in the pattern of alternatively spliced mRNAs (5, 6).

Small molecules can be powerful tools for dissecting complex biological processes, as is well documented for transcription and translation. This is typified by the antibiotics used to study translation in prokaryotes, *e.g.* puromycin and viomycin, which inhibit the prokaryotic ribosome (7). In contrast, there are no comparable classes of natural antibiotics that inhibit the pre-mRNA splicing process. This is likely because natural antibiotics are mostly produced by fungi to kill bacteria, which lack introns in protein-coding genes and thus do not require splicing for protein expression. In recent years, however, several natural compounds, and their synthetic derivatives, have been reported to inhibit splicing, either *in vitro*, or in cells, or both. These include FR901464, spliceostatin A (SSA)^2^ (8), E7107, pladienolide B, pladienolide D (9), GEX1A (10), sudemycin (11), and isoginkgetin (12). With the exception of isoginkgetin, whose target is currently unknown, all the other compounds target the SF3b subunit of the U2 snRNP (13).

Several high throughput screening approaches have also been undertaken to identify small molecules that act as specific splicing inhibitors, albeit with limited success. Most of the molecules that have been identified in these assays either modify splicing in cells but are not shown to do so *in vitro*, *e.g.* clotrimazol, flunarizine, and chlorhexidine (14), or else they inhibit splicing *in vitro* but have not been shown to modulate splicing also in cells, *e.g.* naphthoquinone, tetrocarcin, and ANSC659999 (15).

Two main types of high throughput assay have been established for the identification of new pre-mRNA splicing modifiers as follows: (*a*) cell-based splicing assays, using reporter constructs as a readout (12, 16) and (*b*) high throughput *in vitro* splicing assays, using either ELISA or quantitative PCR techniques for signal detection (15, 17, 18).

The high throughput *in vitro* splicing assay developed by Samatov *et al.* (17) allows the identification of small molecule splicing inhibitors that affect either spliceosome assembly, activation, and/or step 1 catalysis. This is achieved by using the PM5 pre-mRNA, which lacks both the 3′-splice site and 3′ exon of a typical pre-mRNA and therefore stops the splicing reaction after C complex formation. Addition of the PM5 pre-mRNA to a splicing reaction containing FLAG-tagged DDX41, a component of the spliceosomal C complex, allows detection of the C complex using an anti-FLAG antibody. The high throughput screening *in vitro* splicing assay established by Samatov *et al.* (17) thus allows the detection of small molecules that interfere with the splicing process at any stage up to C complex formation. We therefore used this assay to screen a highly selected library of 71,504 small molecules developed by the University of Dundee Drug Discovery Unit to identify new splicing inhibitors (19).

Here, we describe the discovery of 13 new small chemical compounds that inhibit splicing *in vitro*. 10 out of these 13 compounds also inhibit splicing in cultured human cell lines. By way of example, we characterize the effect of one of these compounds, DDD00107587 (madrasin), both on splicing *in vitro* and in cells.

## EXPERIMENTAL PROCEDURES

### 

#### 

##### High Throughput Screening

The high throughput screen was performed using an adapted version of the high throughput *in vitro* splicing assay published by Samatov *et al.* (17) and the in-house small molecule compound library of the Drug Discovery Unit, University of Dundee. In short, using a Wellmate bulk reagent dispenser (Thermo Fisher, Waltham, MA), 384-well polystyrene high protein-binding microplates (Greiner) were coated with rabbit polyclonal antibodies to maltose-binding protein (Abcam, Cambridge, UK) before PM5 pre-mRNA bound by MS2-MBP was added to the microplates and incubated for ∼1 h. 0.1 μl of test compounds were added to the pre-mRNA-labeled microplates by using a Hummingbird (Digilab Genomic Solutions Ltd., Marlborough, MA) to produce a final concentration of 50 μm. The splicing reactions containing HEK293 whole cell extract from cells stably expressing FLAG-tagged DDX41 protein and the monoclonal anti-FLAG® M2-peroxidase (Sigma) were prepared at room temperature, and 20 μl/well were added to the plates using the Biomek FX automated workstation (Beckman Coulter, High Wycombe, UK). The plates were incubated at 26 °C for 90 min before they were washed four times with wash buffer. 20 μm SuperSignal® ELISA Femto Maximum Sensitivity Substrate (Pierce, Thermo Fisher, Rockford, IL) was then added, and the luminescence was monitored after 5 min of incubation at room temperature using the TopCount microplate luminometer (PerkinElmer Life Sciences).

##### Data Analysis

For all primary and potency screen data points, the relative luminescence units per well were measured. From cross-talk-corrected data, a percentage inhibition value for each test compound was determined as follows: % inhibition = 100 −((RLU_TEST_ − RLU_LOW_)/(RLU_HIGH_ − RLU_LOW_)) × 100. RLU_HIGH_ = median of all “high” signal (“uninhibited” control) wells per plate. RLU_LOW_ = median of all “low” signal (noMS2-MBP-pre-mRNA) wells per plate. Robust Z′ was calculated for each plate based on the median and scaled median absolute deviation (1.4826 × median absolute deviation) for RLU_HIGH_ and RLU_LOW_ from all wells per plate. All data processing was conducted within the activity base using normalized data as defined above. Database querying and report creation was undertaken using SARgen version 7.3.0.2 and SARview version 7.1.1 from IDBS (London, UK). All IC_50_ curve fitting was undertaken within the ActivityBase XE utilizing the underlying “Math IQ” engine of XLfit version 5.1.0.0 from IDBS. A four-parameter logistic dose-response curve of the following form was utilized for compound potency determination as shown in Equation 1,

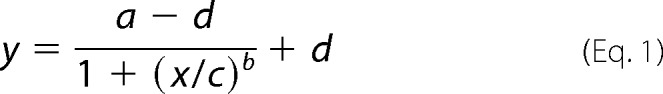
 where *a* is the minimum; *b* is the Hill slope; *c* is the EC_50_ value; and *d* is the maximum. Data are presented as the mean pEC_50_ with the standard error of the mean of *n* experiments.

##### Cell Culture, RNA Isolation, and RT-PCR

HeLa and HEK293 cells were purchased from ATCC and cultured in Dulbecco's modified Eagle's medium supplemented with 10% fetal bovine serum, 2 mm glutamine (Invitrogen), and 100 μg/ml streptomycin (100× stock, Invitrogen). Total RNA was extracted from cells using the NucleoSpin RNA II kit (Macherey-Nagel, Düren, Germany) according to the manufacturer's instructions. 200 ng of total RNA was reverse-transcribed and amplified using the One-step RT-PCR kit (Qiagen, Venlo, Netherlands) according to the manufacturer's instructions. Primer sequences are as follows: *HPV18 E6*, forward 5′-GCGACCCTACAAGCTACCTA-3′ and reverse 5′-GCACTGGCCTCTATAGTGCC-3′ (20); *E1A*, forward 5′-CCGAAGAAATGGCCGCCAGTC-3′ and reverse 5′-GGACGCCGGGTAGGTCTTGC-3′ (21); *RIOK3*, forward 5′-GCTGAAGGACCATTTATTACTGGAG-3′ and reverse 5′-TTCTTGCTGTGTTCTTTCTCCCACA-3′; *Hsp40*, 5′-GAACCAAAATCACTTTCCCCAAGGAAGG-3′ and reverse 5′-AATGAGGTCCCCACGTTTCTCGGGTGT-3′ (22); *BRD2*, 5′-CAAAATTATAAAACAGCCTATGGACATG-3′ and reverse 5′-TTTTCCAGCGTTTGTGCCATTAGGA-3′ (22); *MCL1*, forward 5′-GAGGAGGAGGAGGACGAGTT-3′ and reverse 5′-ACCAGCTCCTACTCCAGCAA; *CCNA2*, forward 5′-TCCTCGTGACTGGTTAGTT-3′ and reverse CCCGTGACTGTGTAGAGTGC-3′; *AURKA*, forward 5′-CTTGGATCAGCTGGAGAGCTT-3′ and reverse 5′-AGCTGATTCTTTGTTTTGGCAAT-3′; *p27* forward 5′-CAGCTTGCCCGAGTTCTACT-3′ and reverse 5′-GTCCATTCCATGAAGTCAGCG-3′.

##### In Vitro Transcription and Nonradioactive in Vitro Splicing Reaction

The adenovirus pre-mRNA Ad1 was transcribed *in vitro* using the RNAMaxx High Yield Transcription kit (Agilent, Santa Clara, CA) according to the manufacturer's instruction, followed by a DNase I digestion and RNA clean up using the RNeasy kit (Qiagen).

Standard splicing reactions were carried out in 30% HeLa nuclear extract (Computer Cell Culture Centre, Seneffe, Belgium) in the presence of either DMSO or compound and incubated at 30 °C for 90 min. The splicing reaction was followed by a heat inactivation step of 5 min at 95 °C before the samples were subjected to proteinase K (Qiagen) digestion for 30 min at 55 °C and another heat inactivation step at 95 °C for 5 min. The spliced and nonspliced RNA was amplified by using the one-step RT-PCR kit (Qiagen) according to the manufacturer's instructions. PCR products were separated by electrophoresis using 1% agarose gels containing SYBR safe DNA gel stain (Invitrogen).

##### Radioactive in Vitro Splicing Reaction and Native Gels

Radioactive *in vitro* splicing reactions were performed as described previously (1) using either ^32^P-labeled pBSAd1 pre-mRNA substrate or ^32^P-labeled MINX pre-mRNA substrate (23). Splicing complexes were analyzed on a low melting point agarose gel (1.5%, w/v) as described previously (24) and visualized by phosphorimaging (Typhoon 8600, GE Healthcare).

##### Cell Fixation and Immunofluorescence Analysis

HeLa cells, treated either with DMSO or compound, were grown on coverslips in DMEM for 24 h at 37 °C before fixing with 4% paraformaldehyde in PHEM buffer for 10 min at room temperature. After rinsing the cells with PBS, the cells were permeabilized with 0.5% Triton X-100 in PBS prior to incubation with the primary antibodies, *i.e.* anti-SC35 (Abcam, Cambridge, UK), anti-coilin (ProteinTech, Chicago, IL), anti-Y12 (Dundee Cell Products, Dundee, UK), anti-TMG (Calbiochem), and anti-SMN (BD Biosciences). After incubation with the primary antibody for 1 h at room temperature, the coverslips were washed twice with 0.5% of Tween 20 in PBS for 5 min before they were incubated with the dye-conjugated secondary antibody for 30 min. Cells were then stained with DAPI (Sigma), and the coverslips were mounted in Vectashield medium (Vector Laboratories, Peterborough, UK). The samples were visualized using a fluorescence microscope (Zeiss, Jena, Germany; Axiovert-DeltaVision Image Restoration; and Applied Precision, LLC).

##### Flow Cytometry Analysis

HeLa cells were seeded in 6-well plates, and after 24 h cells were treated with compound. The cells were harvested after 4, 8, and 24 h and then washed twice with PBS, resuspended in ice-cold 70% ethanol, and fixed at room temperature for 30 min. Fixed cells were then washed twice with PBS, and resuspended in propidium iodide solution (50 μg/ml propidium iodide and 100 μg/ml ribonuclease A in PBS). Cells were incubated in propidium iodide solution for 30 min and then analyzed by flow cytometry on a FACSCalibur (BD Biosciences). The flow cytometry data were analyzed using FlowJo (Treestar Inc., Ashland, OR).

##### Pulse Labeling of HeLa Cells with EdU and EU

Newly synthesized DNA or RNA was detected by using either the Click-iT EdU imaging kit (Invitrogen) or the Click-iT RNA imaging kit (Invitrogen). In brief, HeLa cells were grown for 24 h in the presence of either DMSO or compound and pulse-labeled using either EdU or EU for 20 min. The cells were then fixed, and the Click-iT detection was performed according to the manufacturer's manual.

## RESULTS

### 

#### 

##### High Throughput Screening

**FIGURE 1.**
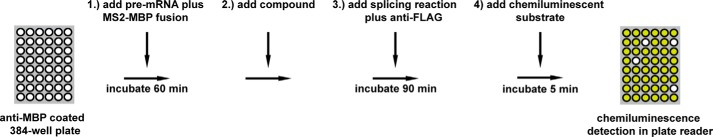
**Schematic representation of *in vitro* splicing high throughput screening.** 384-well plates coated with anti-MBP antibodies were incubated with a mix of pre-mRNA and MS2-MBP fusion protein for 1 h. After addition of 50 μm compound, the splicing reaction containing anti-FLAG antibodies was added, and the splicing reaction was incubated for 90 min at 26 °C. The addition of chemiluminescent substrate for 5 min is followed by signal detection with the TopCount microplate luminometer.

To identify small molecules that inhibit the human spliceosome, we performed a high throughput *in vitro* screen using a splicing assay (17), optimized for robotic screening as outlined in Fig. 1. Thus, the PM5 pre-mRNA was immobilized on anti-MBP antibody-coated 384-well microplates via the MS2-MBP fusion protein. This was incubated in a splicing reaction containing HEK293 whole cell splicing extract, including a FLAG-tagged version of the DEAD box ATPase DDX41. As the PM5 pre-mRNA lacked the 3′ exon and the 3′-splice site, the second step of the splicing reaction could not be performed, and as a consequence, the splicing reaction was stalled after C complex formation. The presence or absence of the C complex on the PM5 pre-mRNA was detected using a horseradish peroxidase-labeled anti-FLAG antibody, and the luminescence was monitored using the TopCount microplate luminometer (PerkinElmer Life Sciences).

A single point screen was performed with a selected library of 71,504 small molecules, each at a final concentration of 50 μm. The test library used a combination of a diverse set (63,874 compounds) and several target-type focused sets, *i.e.* nuclear hormone receptor (2,407), epigenetic modifier (2,664), and kinase (2,559). We chose to follow 352 compounds from the primary screen. This resulted in all molecules giving >52% splicing inhibition being investigated further (Tables 1 and 2).

**TABLE 1:** **Summary of screen statistics (average ± S.D.)**

	Criterion	Result
Valid plates/total plates		217/252
Signal/background	>10	62 ± 37
Robust high control CV (%)	<15	7.2 ± 2.5
Robust Z′	>0.5	0.75 ± 0.08

**TABLE 2:** **Summary of screen outcome**

Output	Result	% total
No. of compounds screened	71,504	
Hit identification threshold	52%	
Putative hits >52%	352	0.49
Putative hits >75%	112	0.16
Putative hits >90%	54	0.07

**FIGURE 2.**
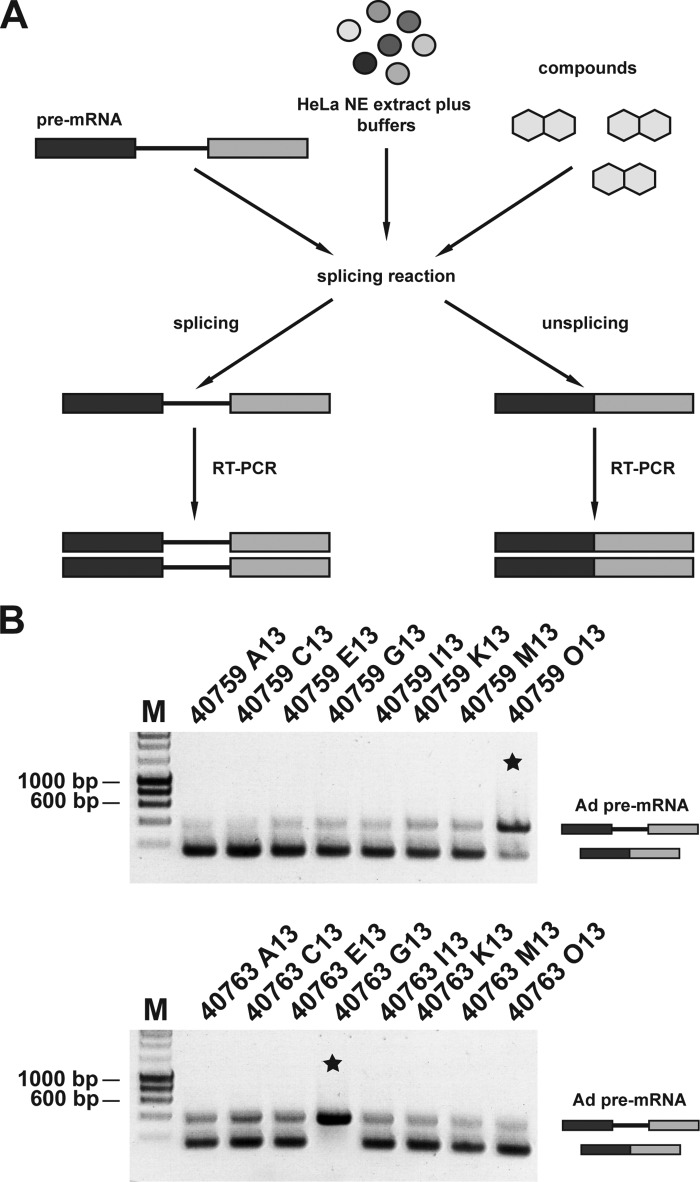
**Secondary screen.** *A,* schematic representation of the RT-PCR *in vitro* splicing assay. 20-μl splicing reaction was incubated in the presence of 500 μm compound at 30 °C for 90 min before the splicing reaction was stop by heat inactivation. After proteinase K digestion, the spliced and unspliced RNA is amplified by RT-PCR. *B* shows examples of compounds that either inhibit pre-mRNA splicing or do not interfere with the splicing reaction. The *star* indicates splicing reactions that were inhibited. *Lane M*, marker (hyperladder, 1 kb).

To reduce false-positive results *e.g.* caused by compounds that restrict the accessibility of the DDX41 antibody to its target, the 352 hit compounds were rescreened using a separate *in vitro* splicing assay format with a nonradioactive RT-PCR-based *in vitro* splicing reaction (Fig. 2). For the secondary screen, a different pre-mRNA, *i.e.* the adenovirus-related Ad1 transcript made from plasmid pBSAd1 (25), was used as a template pre-mRNA, and separate assays were carried out in the presence of (*a*) HeLa nuclear extract and (*b*) HEK293 total cell lysate. A final concentration of 500 μm compound was chosen for this screen to maximize detection of active compounds. The splicing reactions, which were carried out for 90 min, were followed by a proteinase K digestion and a RT-PCR to measure the ratio of spliced/unspliced RNA (see “Experimental Procedures”). This secondary screen reduced the original number of 352 potential hits to 29 molecules that consistently showed inhibition of splicing in each assay format (supplemental Table S1).

DMSO stocks of all 29 compounds were tested by liquid chromatography-mass spectrometry (LC-MS) to confirm the identity, and the composition of each was as expected. 20 of the 29 compounds passed the quality assurance test (supplemental Table S1). For six compounds, the LC-MS test identified a large percentage of impurities, and another three compounds failed due to incorrect molecular weight. The 20 remaining hit compounds were grouped by chemical similarity into six chemical series. Compounds were triaged to remove molecules predicted to be either chemically reactive or unstable. In addition, molecules that have demonstrated frequent activity across a broad range of assays, possibly as a result of being fluorescent, redox active, or aggregating, were removed. One series stood out, however, with three of the most active hit compounds all belonging to a series of aminoquinazolines. These compounds have no obvious undesirable structural features with respect to potential chemical development and have promising physical properties, as represented by DDD00107597, which has a ClogP of 3.3 and a *M*_r_ of 281.

##### Validation of Hit Compounds

Next, we sourced 33 analogues of DDD00107597, both from commercial sources and through in-house synthesis of new compounds. The 33 analogues and DDD00107597 were designed to investigate which structural features are required for splicing modulation. A third round of *in vitro* screening was then performed to confirm the ability of these related compounds to inhibit pre-mRNA splicing. This time the compounds were all tested in two different *in vitro* splicing assays as follows: (*a*) an *in vitro* splicing assay, incubated with HeLa nuclear extract and the radiolabeled adenovirus-derived MINX pre-mRNA (26), and (*b*) a nonradioactive RT-PCR based *in vitro* splicing assay with the Ad1 pre-mRNA, as described above for the secondary screen. Out of this group of 34 compounds, 13 inhibited splicing in at least one assay, with four of these compounds consistently inhibiting pre-mRNA splicing in both assay systems. Of the nine compounds that showed assay-specific splicing inhibition, seven inhibited splicing only in the nonradioactive PCR-based *in vitro* splicing assay using the Ad1 pre-mRNA, and two compounds only inhibited splicing of the MINX pre-mRNA (supplemental Table S2). The reason for these assay-specific inhibitory effects was not clear, and these compounds were not investigated further. We focused instead on the compounds that consistently inhibited splicing in both *in vitro* assay formats.

##### DDD00107587 Inhibits Splicing in Vitro

**FIGURE 3.**
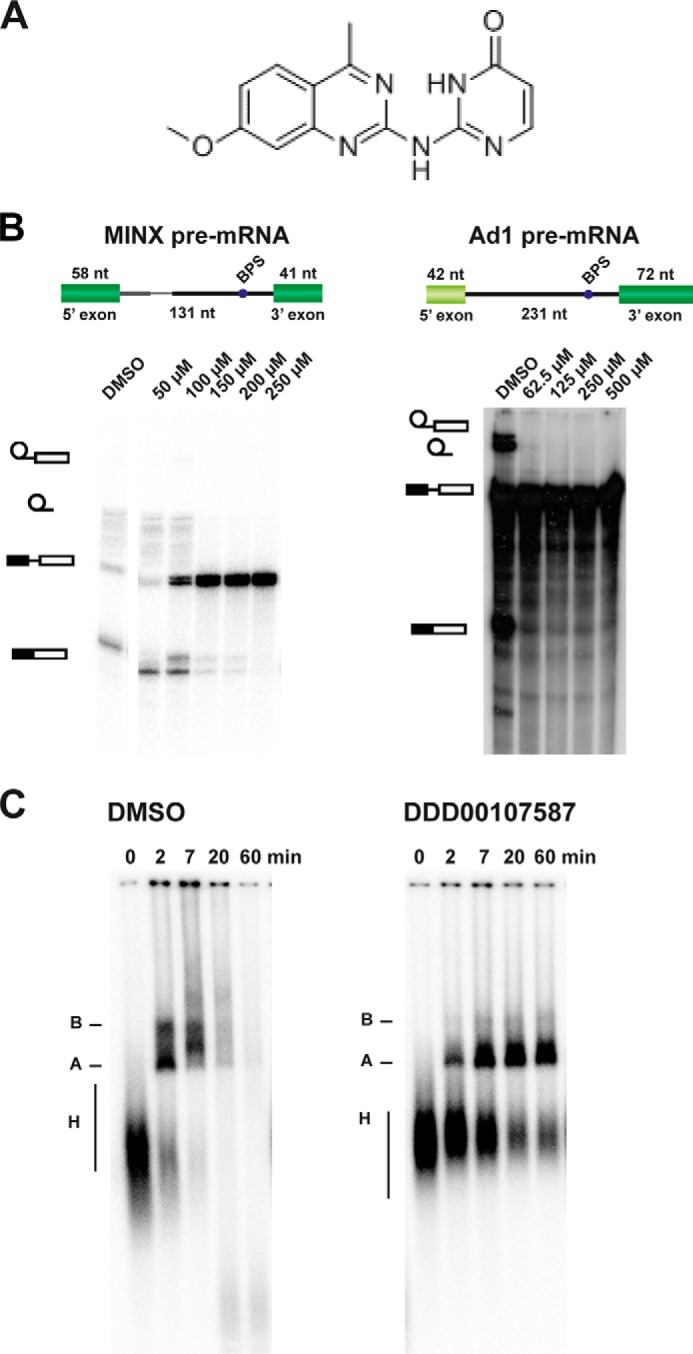
**Effect of DDD00107587 on *in vitro* splicing.** *A,* chemical structure of DDD00107587. *B,* denaturing gel analysis of ^32^P-labeled MINX or Ad1 RNA isolated from splicing reactions incubated with dimethyl sulfoxide (*DMSO*) or different concentrations of DDD00107587. The positions of the lariat intron, lariat, pre-mRNA, and fully spliced mRNA are shown top to bottom on the *left. C,* formation of splicing complex on the MINX pre-mRNA in the presence of DMSO or 250 μm DDD00107587. The reactions were stopped at the time points indicated, and the products were analyzed on a native agarose gel. The positions of the splicing complexes B, A, and H are indicated on the *left*.

Compound DDD00107587 (Fig. 3*A*), one of the four compounds that inhibited splicing of both the MINX and the Ad1 pre-mRNAs (supplemental Table S2), was studied in more detail (Fig. 3). *In vitro* splicing reactions with either radiolabeled MINX or Ad1, pre-mRNAs, were carried out for 2 h in the presence of increasing DDD00107587 concentrations (Fig. 3*B*). Splicing of the Ad1 pre-mRNA was completely inhibited at a final concentration of 62.5 μm, whereas splicing of the MINX pre-mRNA was completely inhibited at 150 μm.

##### DDD00107587 Stalls Spliceosome Assembly at the A Complex Formation

The spliceosome assembles onto pre-mRNA in a stepwise pathway that involves the formation of assembly intermediate sub-complexes. Native agarose gels were used to separate spliceosomes and assemble intermediate complexes to investigate the effect of DDD00107587 on the assembly of splicing complexes on the MINX pre-mRNA. When the splicing assays were carried out in the presence of a DMSO negative control, the native gel shows separation of the H, A, and B complexes. At early times after the start of the splicing reaction (2 and 7 min), mainly A and B complexes are visible. In the presence of 250 μm DDD00107587, which blocks the formation of spliced mRNA, the kinetics of spliceosome assembly differ dramatically. After 2 min, H complexes and small amounts of A complex can be observed. At later times, A complexes accumulate; however, no progression to either the B complex or mature spliceosome was detected (Fig. 3*C*).

These data suggest that DDD00107587 inhibits the formation of spliceosome complexes subsequent to complex A formation. A block of assembly after A complex formation has also been reported for isoginkgetin (12) and spliceostatin A (27). In the case of SSA, however, it was shown that the A complex that forms lacks critical RNA-protein interactions and is therefore relatively instable (28).

##### DDD00107587 Alters Pre-mRNA Splicing in Vivo

Next, we investigated the effect of DDD00107587 on the splicing of endogenous pre-mRNAs *in vivo*. HeLa and HEK293 cells were each treated with either DMSO, as a negative control, or with DDD00107587 at a final concentration of 10, 20, or 30 μm, for either 4, 8, or 24 h. After incubation, the cells were harvested, and total RNA was extracted. 200 ng of total RNA was used in RT-PCR assays to detect both intron inclusion in transcripts from the *RIOK3*, *BRD2*, and *Hsp40* genes and changes in the alternative splicing of the *MCL1*, *CCNA2*, *AURKA*, and *p27* pre-mRNAs. In addition, primers were chosen to detect the splicing of the *HPV18 E6* (human papillomavirus type 18) RNA in HeLa cells and the adenovirus *E1A* RNA in HEK293 cells.

**FIGURE 4.**
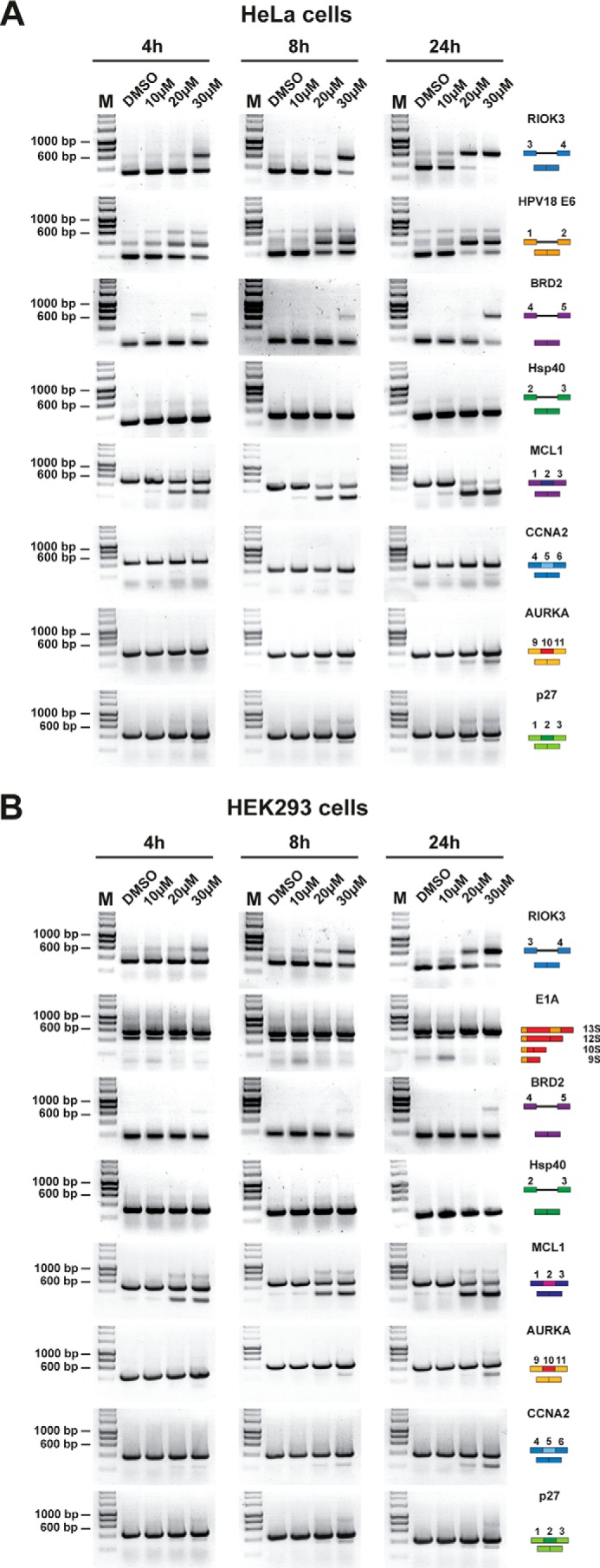
**Splicing inhibition in cells treated with DDD00107587.** Semiquantitative RT-PCR analysis of cells treated with increasing concentrations of DDD00107587 and DMSO for 4, 8, and 24 h. HeLa cells were investigated for intron inclusion of *RIOK3*, *HPV18 E6*, *BRD2*, and *Hsp40* as well as the exon skipping of *MCL1*, *AURKA*, *CCNA2*, and *p27* (*A*). HEK293 cells were examined for intron inclusion of *RIOK3*, *BRD2*, and *Hsp40*, the alternative splicing the adenovirus *E1A* gene and the exon skipping of *MCL1*, *AURKA*, *CCNA2*, and *p27* (*B*). The positions of different cDNA products are pictured on the *right* of each gel image, and molecular weight markers are shown on the *left. Lane M*, marker (hyperladder, 1 kb).

Fig. 4*A* shows the effect on pre-mRNA splicing changes induced by DDD00107587 in HeLa cells. Although no splicing inhibition of the *Hsp40* pre-mRNA could be detected at the concentrations and time points tested, inhibition of splicing of each of the *RIOK3*, *HPV18*, and *BRD2* pre-mRNAs was observed after 4 h of treatment at 30 μm. Only a slight increase in inhibition of pre-mRNA splicing over time was seen for most *HPV18 E6* and *BRD2* pre-mRNAs. DDD00107587 potently inhibited splicing of the *RIOK3* pre-mRNA, with >95% splicing inhibition at 30 μm for 24 h and ∼80% splicing inhibition at 20 μm for 24 h.

Exon skipping after DDD00107587 treatment was observed for all four genes (*MCL1, CCNA2, AURKA*, and *p27*) examined. The strongest effect was observed on exon 2 skipping of the *MCL1* pre-mRNA. 4 h after addition of DDD00107587 to HeLa cells, a significant shift from the long splice variant of *MCL1* (MCL1L) to the short splice variant (MCL1S) was observed at concentrations of 20 and 30 μm. Over time this shift increased until almost exclusively the short isoform was expressed at these concentrations. At 10 μm, low levels of MCL1S were detected, which did not increase over time. Weak exon skipping was observed for *AURKA* and *p27* after 4 h at concentrations of 20 and 30 μm DDD00107587. Changes in alternative splicing of *AURKA* and *p27* increased slightly over time. DDD00107587 treatment resulted only in low amounts of alternatively spliced *CCNA2* transcripts after 24 h at 30 μm.

The results for HEK293 cells are very similar to those in HeLa cells (Fig. 4*B*), with little or no pre-mRNA splicing inhibition detected in the case of the *Hsp40* pre-mRNA, but clear inhibition of splicing of the *RIOK3* and *BRD2* pre-mRNAs was detected 24 h after treatment at a final concentration of 30 μm. The alternative splicing of the *E1A* gene was also changed in the presence of DDD00107587 in a dose-dependent manner. Whereas treatment of HEK293 cells with 10 μm DDD00107587 led to a shift toward formation of the 9 S splice variant, 20 and 30 μm DDD00107587 resulted in the accumulation of 13 S splice products. In HEK293 cells, exon skipping for the four transcripts tested was only detected for the two highest concentrations of DDD00107587, 20 and 30 μm. As in HeLa cells, DDD00107587 had the greatest effect on the alternative splicing of *MCL1* with significantly more MCL1S expressed than MCL1L after 24 h of treatment. Exon skipping of *AURKA*, *CCNA2*, and *p27* was mostly observed at 30 μm.

##### DDD00107587 Causes Cell Cycle Arrest

**FIGURE 5.**
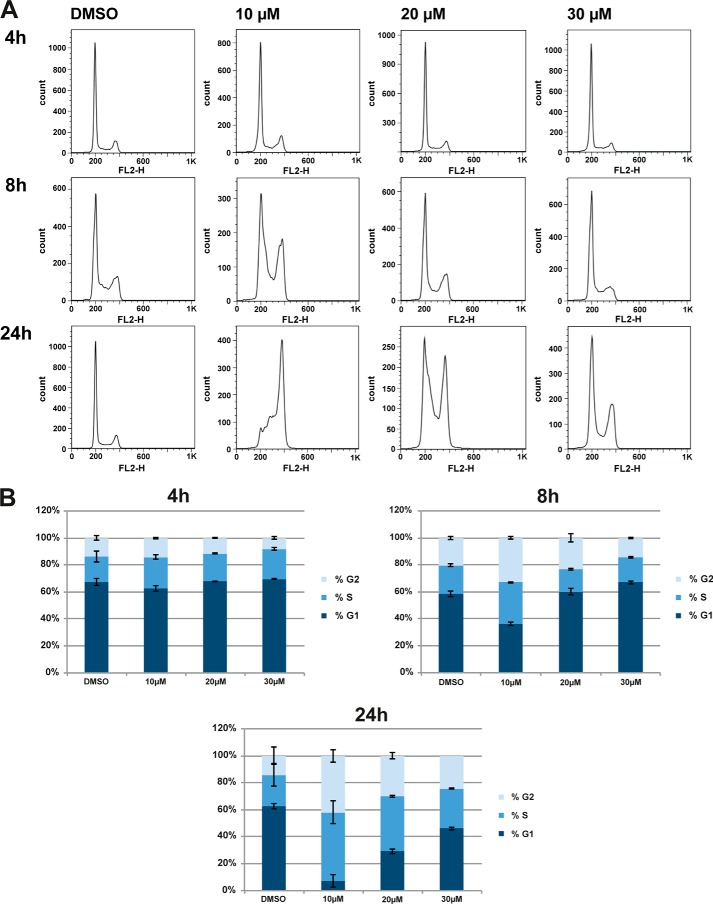
**DDD00107587 induces S/G_2_ and M phase arrest.** Cell cycle analysis was performed on HeLa cells treated with DMSO and 10, 20, and 30 μm DDD00107587 for 4, 8, and 24 h. Cellular DNA content was measured by propidium iodide staining followed by flow cytometry analysis. *A,* histograms of DNA content. *B,* cell cycle distributions quantified using the Watson model (51).

We next tested the effect of DDD00107587 on cell cycle progression in exponentially dividing cells. HeLa cells were treated either with a DMSO negative control or with a final concentration of 10, 20, or 30 μm DDD00107587 for 4, 8, and 24 h before labeling with propidium iodide and analysis by flow cytometry (Fig. 5*A*). No cell cycle changes were observed after 4 h of treatment at any concentration tested. In the presence of 10 μm DDD00107587 8 h after treatment, the proportion of cells in G_2_, M, and S phases increased, with a concomitant decrease in the number of G_1_ phase cells. This effect increased over time, with >40% of cells in G_2_ and M phase and >50% in S phase 24 h after treatment with DDD00107587. This contrasted with 14% in G_2_ and M and 23% in S phase in the DMSO control (Fig. 5*B*). Cells treated with higher concentrations showed a delayed cell cycle arrest. 8 h of treatment with either 20 or 30 μm DDD00107587 exhibited the same cell cycle profile as the DMSO control. At 24 h, however, cells treated with 20 or 30 μm DDD00107587 also showed G_2_, M, and S phase arrest, with ∼40% of cells in S and ∼30% in G_2_ and M phase in the presence of 20 μm and 30% of cells in S and 24% in G_2_ and M phase when treated with 30 μm DDD00107587.

**FIGURE 6.**
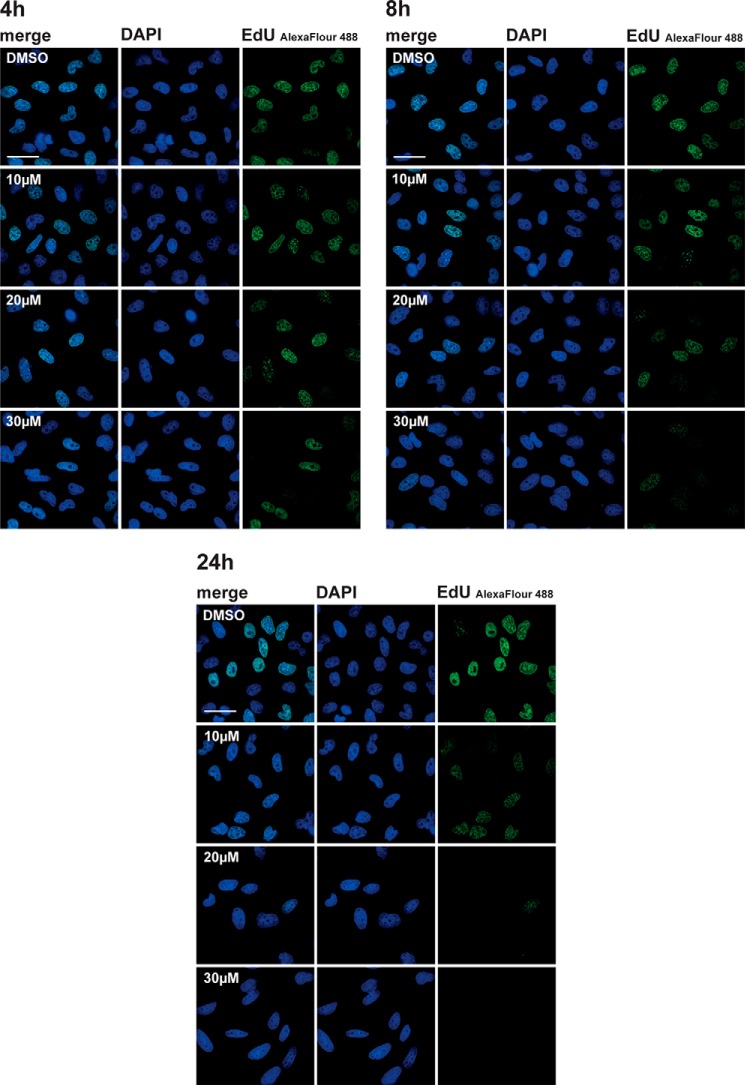
**DDD00107587 reduces DNA synthesis.** HeLa cells were treated with DMSO and 10, 20, and 30 μm DDD00107587 for 4, 8, and 24 h. Areas of active DNA replication were marked by pulse labeling with EdU 20 min before the cells were fixed. *Scale bars,* 40 μm.

To test whether cells arrested by DDD000107587 showed any inhibition of DNA synthesis, HeLa cells were pulse-labeled with EdU either 4, 8, or 24 h after treatment with different concentrations of DDD000107587 (Fig. 6). DDD00107587 showed a time- and dose-dependent effect on reducing DNA synthesis, with the greatest reduction in DNA synthesis at 30 μm. After 4 h of treatment with 30 μm DDD00107587, a modest reduction in DNA synthesis was detected (∼20%), which increased over time until after 24 h no incorporation of EdU could be detected at this concentration. Reduction in DNA synthesis levels in HeLa cells was first observed after 8 h of treatment with 20 μm, with essentially complete inhibition after 24 h. At the lowest concentration tested, 10 μm, a decrease in DNA synthesis could only be detected after 24 h.

**FIGURE 7.**
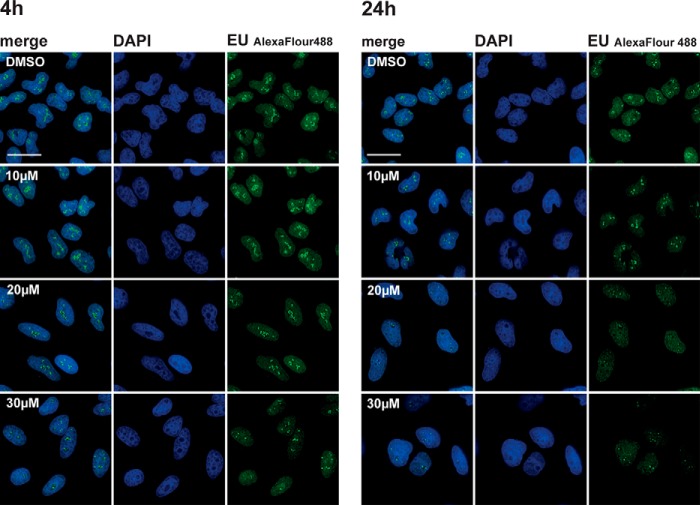
**DDD00107587 does not inhibit transcription of RNA polymerase II.** RNA synthesis in HeLa cells treated with DMSO or 10, 20, and 30 μm for either 4 or 24 h was analyzed by EU pulse labeling. *Scale bars,* 30 μm.

Next, we tested whether DDD00107587 had a similar inhibitory effect on levels of newly transcribed RNA in HeLa cells by pulse labeling with EU (see “Experimental Procedures”). In contrast with the reduced levels of DNA synthesis, total RNA synthesis levels appeared less affected by DDD00107587 (Fig. 7). In particular, nucleoplasmic transcription levels showed minimal reduction, consistent with the RT-PCR data (Fig. 4) showing an accumulation of unspliced pre-mRNA. Interestingly, the main inhibitory effect on nuclear RNA caused by DDD00107587 was a decrease in nucleolar RNA levels, likely representing decreased rRNA synthesis (Fig. 7).

##### Loss of Cajal Bodies after Treatment with DDD00107587

**FIGURE 8.**
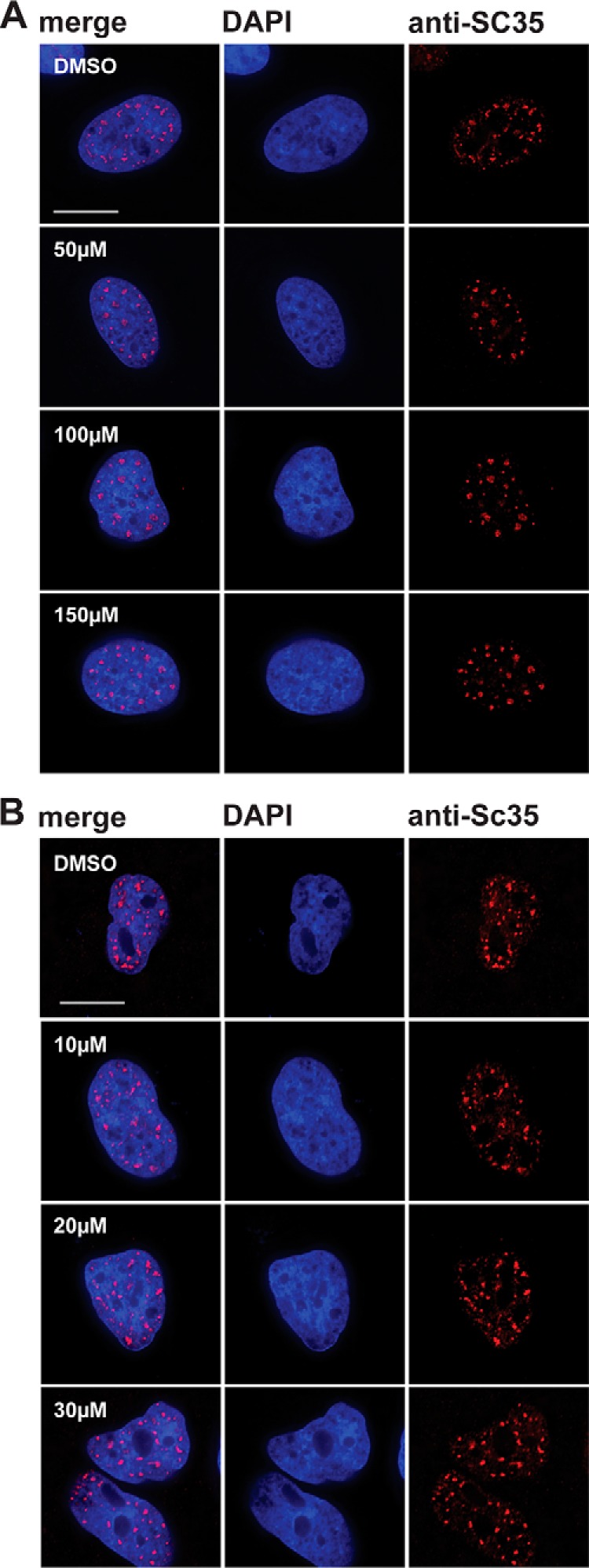
**Splicing speckles morphology is not changed by treatment with DDD00107587.** SC35 immunofluorescence of HeLa cells treated with DMSO or 10, 20, and 30 μm DDD00107587 (*B*) and 50, 100, and 150 μm DRB (*A*) for 4 h. *Scale bars,* 15 μm.

**FIGURE 9.**
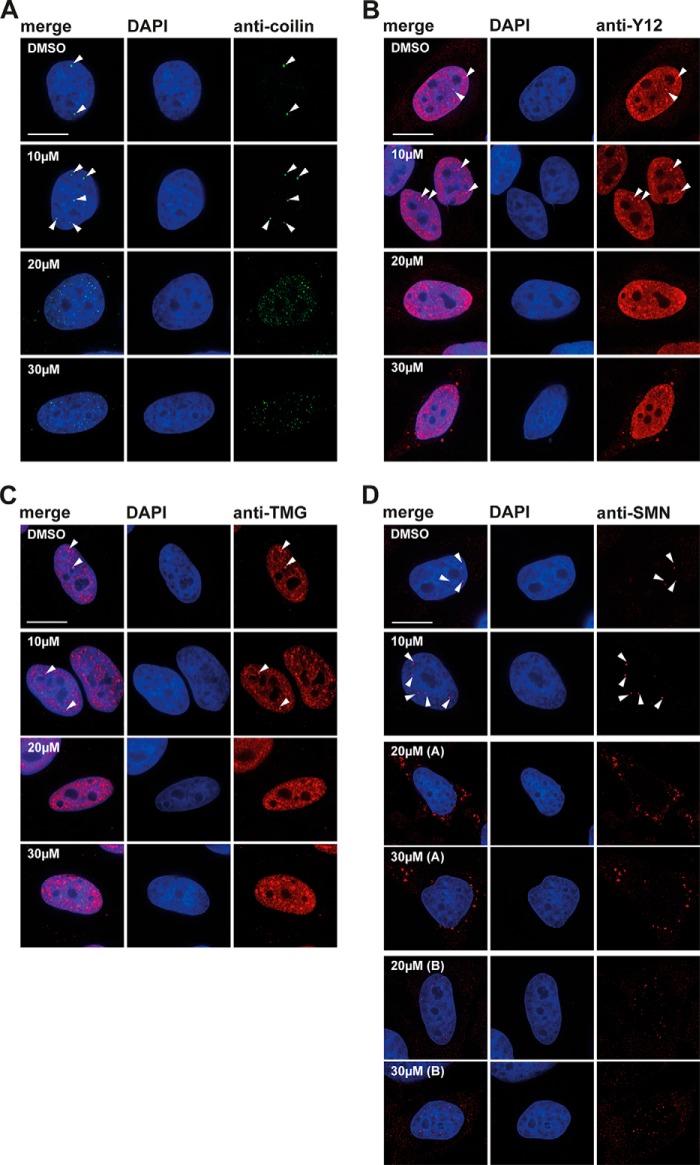
**Cajal body disruption after treatment with DDD00107587.** HeLa cells treated with DMSO or 10, 20, and 30 μm for 4 h were stained with anti-coilin (*A*), anti-Y12 (*B*), anti-TMG (*C*), and anti-SMN (*D*) antibodies, respectively. *Arrowheads* denote intact CBs. *Scale bars,* 15 μm.

**FIGURE 10.**
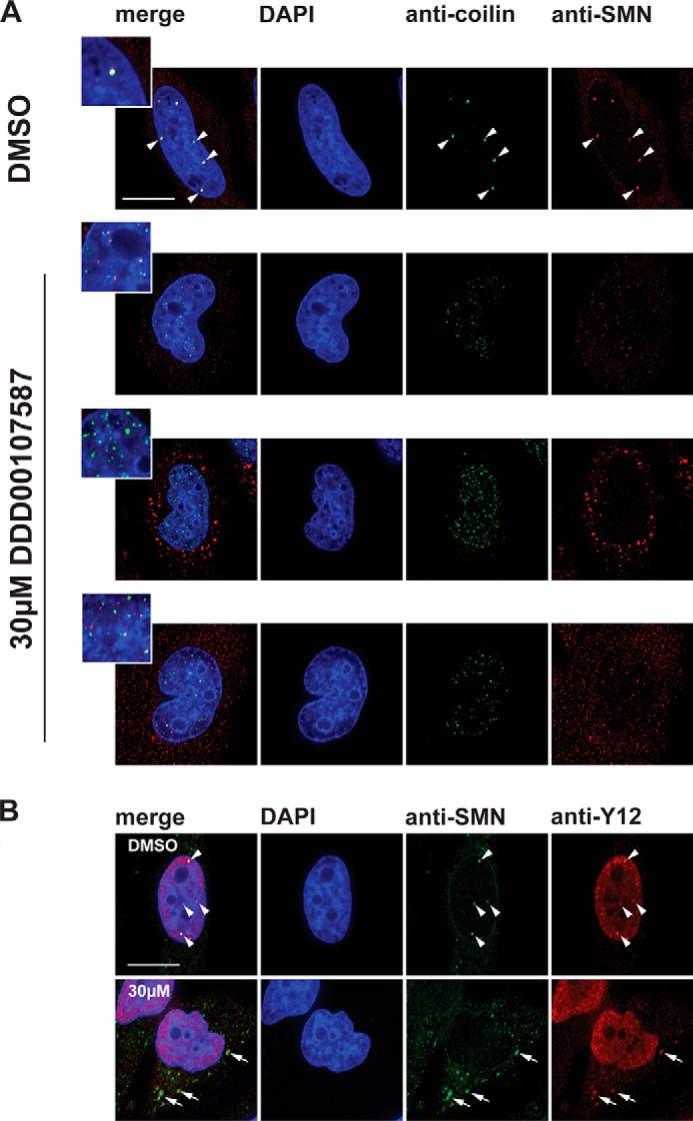
**Subcellular localization of SMN, coilin, and Y12 after treatment with DDD00107587.** HeLa cells were treated for 4 h with DMSO or 30 μm DDD00107587. The localization of SMN and coilin (*A*) as well as SMN and Y12 (*B*) was visualized by immunostaining. *Arrows* mark the colocalization of cytoplasmic SMN and Y12 aggregates in the presence of DDD00107587. *Arrowheads* denote intact CBs. *Scale bars,* 15 μm.

Next, we investigated the effect of DDD00107587 on subnuclear structures and specifically on the organization of pre-mRNA splicing factors within the nucleus. To analyze the effect of DDD00107587 on splicing speckles, HeLa cells were treated for 4 h with either a DMSO negative control or with 10, 20, or 30 μm DDD00107587 before cells were fixed and stained with an antibody to label the splicing factor SC35 (Fig. 8). Interestingly, no enlargement and rounding up of the speckles was detected (Fig. 8*A*), in contrast to what is typically observed when transcription is inhibited (Fig. 8*B*). However, DDD00107587 induced a dose-dependent disruption of CBs. Thus, antibodies to each of four CB markers (coilin, SMN, TMG cap, and Y12) label bright foci, corresponding to CBs (Fig. 9, *arrowheads*), in cells treated with the DMSO control. Intact CBs were still visible when HeLa cells were treated for 4 h with 10 μm DDD00107587. However, after 4 h of treatment with either 20 or 30 μm DDD00107587, the bright CB foci were no longer observed, and coilin instead forms numerous microdots throughout the nucleoplasm (Fig. 9*A*). This change in coilin localization was consistently observed in essentially all cells, whereas a subset of these cells also showed SMN forming a multitude of tiny dots in the nucleoplasm (Fig. 9*D*). Other cells treated for 4 h with either 20 or 30 μm DDD00107587 displayed instead large SMN aggregates in the cytoplasm (Fig. 9*D*). The coilin microfoci mostly did not colocalize with SMN and never colocalized in cells showing SMN aggregates in the cytoplasm (Fig. 10*A*). In contrast with coilin and SMN, Y12 and TMG are not only located in CBs but also in nuclear speckles. After treatment for 4 h with either 20 or 30 μm DDD00107587, both the Y12 and TMG antibodies no longer stained CBs, although both still labeled a similar pattern of speckles compared with the DMSO control. Furthermore, the morphology of the splicing speckles showed little or no change (Fig. 9, *B* and *C*). In the case of Y12, some cells also showed cytoplasmic aggregates in the presence of 30 μm DDD00107587 for 4 h. These Y12 aggregates colocalize with the cytoplasmic SMN aggregates (Fig. 10*B, arrows*).

**FIGURE 11.**
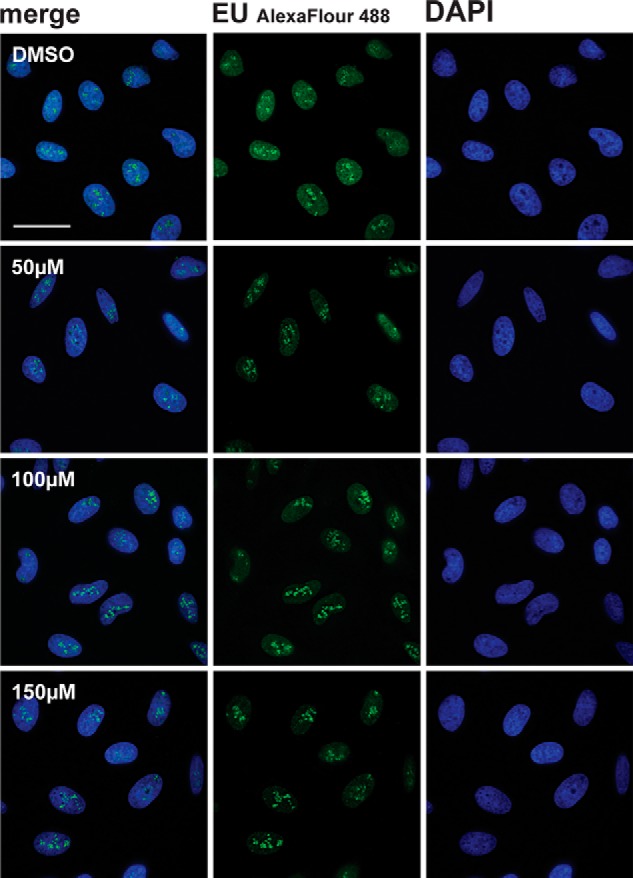
**DRB inhibits transcription of RNA polymerase II.** HeLa cells were incubated for 4 h with DRB before the cells were incubated with EU that was incorporated into newly synthesized RNA for 20 min. Cells were then fixed, and labeled RNA was detected with the help of a fluorescent microscope. *Scale bar,* 40 μm.

**FIGURE 12.**
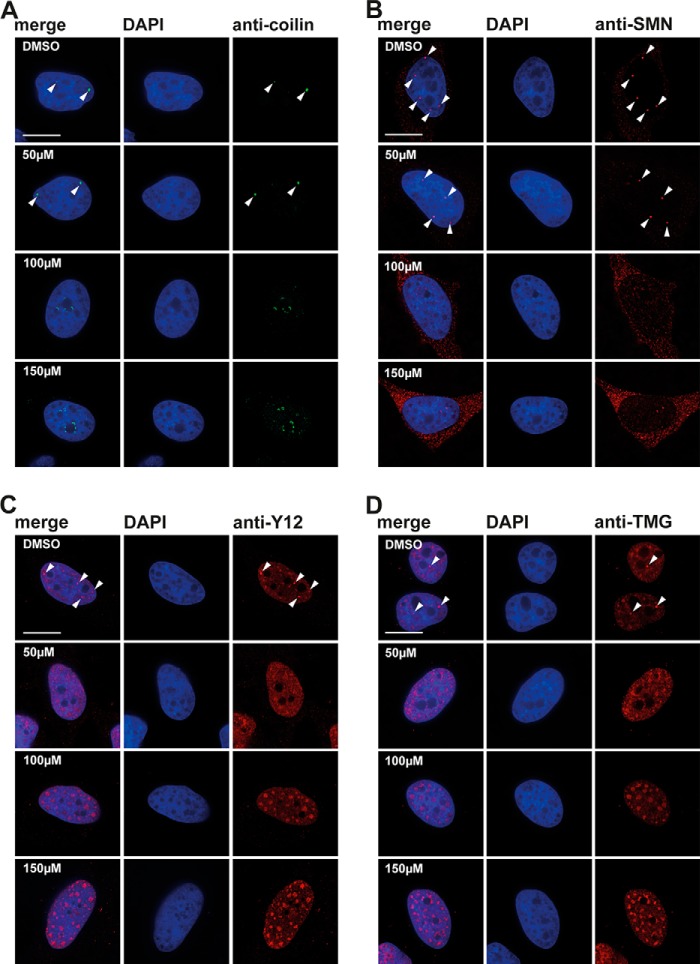
**Cajal body disruption after treatment with DRB.** HeLa cells were incubated with DMSO or 50, 100, and 150 μm DRB for 4 h and stained with anti-coilin (*A*), anti-SMN (*B*), anti-Y12 (*C*), and anti-TMG (*D*) antibodies respectively. *Arrowheads* denote intact CBs. *Scale bar,* 15 μm.

As it is known that RNA polymerase II activity is required for CB integrity, we next compared the effect of DDD00107587 on the redistribution of CB components with that induced by treating cells with transcription inhibitors. HeLa cells were treated with either 50, 100, or 150 μm DRB for 4 h to block RNA polymerase II (Fig. 11). This showed that the CB disruption in response to DRB treatment is different from that induced by DDD00107587. Thus, the staining with both the Y12 and anti-TMG antibodies show that in the presence of either 100 or 150 μm DRB for 4 h, the nuclear speckles become more rounded and increase in size (Fig. 12, *C* and *D*). Coilin and SMN were still visible in foci after 4 h of incubation with 50 μm DRB, but they no longer colocalized with snRNP antigens Y12 and TMG. After the total disruption of CBs caused by 4 h of treatment with either 100 or 150 μm DRB, coilin relocated into nucleolar caps (Fig. 12*A*), as observed previously (29), whereas SMN could mainly be detected in a diffuse cytoplasmic pool (Fig. 12*B*).

## DISCUSSION

Using a recently described ELISA-based high throughput screening *in vitro* splicing assay (17), we screened a diverse small molecule library of 71,504 compounds. This primary screen was followed with independent, secondary screens using a combination of different *in vitro* splicing assay formats. We have thereby identified new small molecule splicing inhibitors, which are active *in vitro* and in cells. These small molecule splicing inhibitors are structurally distinct from previously described natural compound splicing inhibitors. One of these new splicing inhibitors, DDD00107587 (2-((7methoxy-4-methylquinazolin-2-yl)amino)-5,6-dimethylpyrimidin-4(3*H*)-one, was characterized in more detail with respect to its effect on spliceosome assembly and nuclear structure. For practical reasons we have renamed DDD00107587 as madrasin (2-((7methoxy-4-methylquinazolin-2-yl)amino)-5,6-dimethylpyrimidin-4(3*H*)-oneRNAsplicing inhibitor).

Several different classes of compounds have been reported to inhibit splicing *in vitro* and/or to modify alternative splicing of specific pre-mRNA transcripts in cells. These compounds include acetylation/deacetylation inhibitors (23), kinase inhibitors (23, 30), phosphatase inhibitors (31), topoisomerase I inhibitors (32), and cardiotonic steroids (16, 33). However, very few natural compounds and their synthetic derivatives have been reported to inhibit splicing both *in vitro* and *in vivo e.g.* FR901464 (8), GEX1A (10), pladienolide B, pladienolide D (9), and isogengketin (12).

Madrasin is not related in structure to these previously described inhibitors. It prevents formation of both splicing intermediates and products *in vitro* and interferes with one or more early steps in the pathway of spliceosome assembly, allowing the formation of the A complex but blocking the formation of larger spliceosome complexes. Other previously described splicing inhibitors have also been shown to target early events in the spliceosome assembly pathway, resulting from their having the same target, *i.e.* SF3b. This is a multiprotein subunit of the U2 snRNP that is present in the A complex. For example, SSA and pladienolide (27) both stall spliceosome assembly after A complex formation. However, despite having the same target SF3b, meayamycin has been reported to stop the splicing process after E complex formation (34). To date, the molecular target has only been identified for the splicing modulators isolated from the broth of bacteria, *e.g.* FR901464, pladienolide, and GX1A and their derivatives, which all bind the SF3b subcomplex of U2 snRNP.

Splicing inhibition has long been associated with cell cycle arrest. Not only has the down-regulation of splicing factors such as SFRS1, SFRS2, and U2AF35 (35–37) by siRNA depletion been shown to arrest cells in G_2_ and M, but treatment with splicing inhibitors such as SSA (HeLa cells), GEX1A (WI-38 cells), and E7107, as well as pladienolide (WiDr cells), all arrest cells in G_1_, G_2_, and M phase (8, 38–40). The cell cycle arrest caused by the inhibitors shown to target SF3b has been mainly attributed to the inhibition of splicing of the *p27* pre-mRNA. p27 is a cyclin-dependent kinase inhibitor important for the regulation of cell cycle progression. Altered *p27* splicing results in the expression of a C-terminally truncated version of the p27 protein (8). Recently, the effect of SSA on splicing was studied in more detail using a splicing-sensitive microarray, which showed that in addition to inhibition of *p27* splicing, the splicing of other genes important for cell cycle progression, including *CCNA2* and *AURKA*, was also affected (28).

We demonstrate here that madrasin shows a dose- and time-dependent inhibitory effect on cell cycle progression in HeLa cells. Low doses (10 μm) of madrasin show a clear accumulation of cells in S, G_2_, and M phases after 8 and 24 h of treatment. Higher doses of madrasin also result in the accumulation of cells arrested in S, G_2_, and M. However, the cell cycle arrest at 20 and 30 μm madrasin could only be detected after 24 h, which suggests a block in the transition of all cell cycle stages. Our data indicate that the cell cycle arrest induced by 10 μm madrasin might be different from that observed for the two higher concentrations. Like the SF3b targeting compounds, madrasin also modulates the alternative splicing of *MCL1*, *CCNA2*, *AURKA*, and *p27*. However, exon skipping of these genes was almost exclusively detected at 20 and 30 μm and therefore is unlikely to account for the cell cycle arrest induced by 10 μm madrasin. The cell cycle arrest seen with the higher concentrations coincides with a reduction in DNA synthesis levels as shown by the reduced incorporation of EdU in cells treated with 20 and 30 μm madrasin for 8 h. In contrast, a reduction in DNA synthesis, as judged by reduced EdU incorporation, was only detected after 24 h of treatment, whereas cell cycle arrest was observed after 8 h in the presence of 10 μm madrasin. Even though inhibition of DNA synthesis caused either by DNA damage or by the depletion of deoxyribonucleotide precursors is a well known cause of cell cycle arrest, it is likely that the cell cycle arrest caused by madrasin results from altered splicing of one or more transcripts that encode proteins needed for normal cell cycle progression. Therefore, the observed inhibition of DNA synthesis is more likely an indirect effect.

It has been previously reported that either treatment of cells with splicing inhibitors, *e.g.* SSA, or the down-regulation of gene expression caused by transcription inhibitors, *e.g.* DRB, changes the morphology of splicing speckles, which become round and enlarged (41). Splicing speckles are granules located in interchromatin regions, which are enriched in splicing factors (41). Treatment with madrasin caused little or no reduction in RNA polymerase II transcription, as judged by nucleoplasmic EU incorporation in HeLa cells. In agreement with these findings, madrasin treatment also did not result in morphological changes of nuclear speckles. However, treatment with madrasin did result in the disruption of Cajal bodies in HeLa cells.

In contrast to polymerase II transcription, a noticeable reduction in newly synthesized rRNA was observed after 24 h of incubation with madrasin. A possibility for this decrease in rRNA synthesis might be indirect effects caused by splicing inhibition. Besides the down-regulation of factors important for rRNA transcription, splicing inhibition might also cause a decrease in the production of small nucleolar RNAs. Small nucleolar RNAs are essential for the maturation of rRNA in the nucleolus and most small nucleolar RNAs in mammalian cells are located in introns and processed from spliced introns (42).

CBs play an important role not only in the maturation of small nucleolar RNAs, small Cajal body-specific RNAs, and telomerase RNAs but also in the maturation of the spliceosomal snRNPs (43). It is known that the integrity of CBs depends on ongoing RNA synthesis, and inhibition of transcription leads to the disruption of CBs (41). As RNA polymerase II transcription is not prevented by madrasin treatment, another mechanism must be responsible for causing the loss of CBs. Redistribution of coilin throughout the nucleus independently of transcription inhibition has been described after up-regulation of PA28γ (29). Another difference between the disruption of CBs caused by transcription inhibition and by madrasin treatment is the effect on coilin localization. Coilin relocates to nucleolar caps after transcription inhibition, but not, as shown here, after madrasin treatment, when coilin relocates to form numerous microfoci throughout the nucleoplasm.

During the cell cycle CBs disassemble in M phase and reappear as multiple small foci in early G_1_ phase. It is unlikely that the changes seen in the localization of coilin after madrasin treatment are indirectly due to changes in the cell cycle, however, as no cell cycle arrest was observed after 4 h treatment with madrasin by which time CB disruption and changes in coilin localization were present in all cells. In addition, the nuclear localization of other CB components analyzed also behave differently after treatment of cells with madrasin, as compared with transcription inhibition. For example, after transcription inhibition, the snRNPs detected with the Y12 antibody are located in enlarged, rounded splicing speckles, whereas Y12 instead labels cytoplasmic aggregates, as well as morphologically normal splicing speckles, after a 4 h treatment of HeLa cells with 30 μm madrasin (Fig. 9). To date, none of the previously described molecules that alter splicing have been shown to cause a similar disruption of CBs. Therefore, madrasin provides a unique chemical tool with which to study the relationship between splicing and CBs in greater detail.

Interestingly, madrasin does not inhibit splicing of all transcripts we tested *in vivo*. For example, pre-mRNA splicing of the *Hsp40* pre-mRNA was relatively unaffected by the same concentration and time of treatment with madrasin that strongly altered the splicing of RIOK3. It was previously assumed that general splicing inhibitors, like SSA, which target the U2 snRNP splicing factor SF3b 155, would likely cause inhibition of all pre-mRNA splicing, because U2 snRNP is thought to be needed for splicing of all GT-AG introns. However Corrionero *et al.* (28) showed recently using splicing arrays to examine global splicing events that, instead of affecting all transcripts, SSA alters splicing of only a subset of genes, 3 h after treatment. To analyze the full extent of the splicing changes induced by madrasin and compare this with the effects of other splicing inhibitors, it will be important in the future to assess also changes in global transcription levels and alternative splicing patterns.

Our results highlight the potential of madrasin as a tool that can be used to dissect the splicing mechanism *in vitro* and in cells. However, as madrasin is not a natural product, rather it was identified from a library of drug-like small molecules, its further development may also prove useful in understanding the therapeutic potential of splicing inhibition. Increasingly, pre-mRNA splicing is becoming an attractive target for drug discovery (44–47). Splicing inhibitors, including meamyacin (34), isoginkgetin (48), and pladienolides (49), have been shown to have anticancer properties. In the case of pladienolides, it has been demonstrated that the targeting of SF3b is the direct mechanism of their antitumor activity (40). Most recently, while this manuscript was under review, Naryshkin *et al.* (50) have reported the identification of small molecules that alter the splicing of transcripts from the *SMN2* gene, which is linked with genetic disease causing neuromuscular degeneration and mortality. Consistent with the known effects of other splicing inhibitors, madrasin exhibits cytostatic activity. In addition, madrasin also alters the splicing of at least two viral oncogenes, *i.e. E1A* (adenovirus) and the *HPV18 E6* (human papillomavirus type 18), which may be interesting with regard to the effects of madrasin-related compounds on viral reproduction and their potential to transform cells.

In summary, we have identified a new class of small molecule splicing modulators that inhibit splicing of multiple introns both *in vitro* and in cells. Further characterization of this compound, and its related derivatives, may lead to the development of more potent and transcript-selective compounds with uses in studying the splicing mechanism and potential applications as antiviral and/or anti-cancer agents.

## Supplementary Material

Supplemental Data

## Footnotes

2 The abbreviations used are:

SSA: spliceostatin A
snRNP: small nuclear ribonucleoprotein
CB: Cajal Body
EU: 5-ethynyl uridine
EdU: 5-ethynyl-2′-deoxyuridine
DRB: 5,6-dichloro-1-β-d-ribofuranosylbenzimidazole
MBP: maltose-binding protein
RLU: relative luminescence unit
SMN: survival of motor neuron 1, telomeric.
