# Supplementary material for: Identification of Small Molecule Inhibitors of Pre-mRNA Splicing
Source: J Biol Chem. 2014 Oct 3;289(50):34683–98. doi: 10.1074/jbc.M114.590976 (PMC4263873; doi:10.1074/jbc.M114.590976)
Supplement: Supplemental Data [file supp_289_50_34683__index.html]

Identification of Small Molecule Inhibitors of Pre-mRNA Splicing — Identification of Small Molecule Inhibitors of Pre-mRNA Splicing — Identification of New Small Molecule Splicing Inhibitors — Supplemental Data 

# Identification of Small Molecule Inhibitors of Pre-mRNA Splicing

## Supplemental Data

**Files in this Data Supplement:**

- Supplemental Table 1 (.xlsx, 1.4 MB) - LC-MS results of compounds identified as potential splicing modulators in a secondary screen
- Supplemental Table 2 (.xlsx, 93 KB) - Chemical structures of DDD00107597 and its analogues and the effect of these compounds on *in vitro* splicing
